# The genome of *Phlebotomus chinensis*, the primary vector of visceral leishmaniasis in China: insights from chromosome-level assembly and comparative analysis

**DOI:** 10.1186/s40249-026-01417-w

**Published:** 2026-02-06

**Authors:** Haowei Dong, Wenqi Shan, Qiuming Zhou, Hao Yuan, Kang Wang, Yuanyuan Li, Wenbing Zhong, Maimaitijiang Wumaier, Anjie Yang, Huiying Chen, Bing Rui, Yajun Ma, Shizhu Li, Heng Peng

**Affiliations:** 1 Department of Pathogen Biology, College of Basic Medicine, Naval Medical University, Shanghai, 200433 China; 2 Present Address: Key Laboratory of Biosafety Defense, Ministry of Education, Beijing, China; 3 Present Address: Faculty of Naval Medicine, Naval Medical University, Shanghai, 200433 China; 4 Yangquan Center for Disease Control and Prevention, Yangquan, 045000 Shanxi China; 5 Chinese Center for Disease Control and Prevention, National Institute of Parasitic Diseases, Shanghai, 200025 China; 6 Haikou Center for Disease Control and Prevention, Haikou, 570203 Hainan China; 7 Institute of Parasitic and Brucellosis Prevention and Treatment, Center for Disease Control and Prevention of Xinjiang Uygur Autonomous Region, Urumqi, 830002 China

**Keywords:** *Phlebotomus chinensis*, Genome, Third-generation sequencing, Leishmaniasis, China

## Abstract

**Background:**

*Phlebotomus chinensis* is the primary vector of visceral leishmaniasis (VL) in China. However, the lack of a high-quality genome assembly for this species has limited research on its biology, vector-pathogen interactions, and evolutionary adaptations. To address this critical gap, the first chromosome-level genome assembly of *Ph. chinensis* was constructed.

**Methods:**

Nanopore long-read sequencing served as the primary method, complemented by Illumina short-read sequencing for base-level error correction and Hi-C mapping for chromosomal anchoring and chromosome-level scaffolding. Genome annotation integrated transcriptome data from adult, larvae and pupae, homologous protein predictions from closely related sand fly species, and ab initio gene prediction. Comparative genomic analyses were further performed to explore evolutionary relationships and genomic differences between *Ph. chinensis*, *Ph. papatasi*, and *Lutzomyia longipalpis*.

**Results:**

A total of 127.05 Gb of Nanopore data, 10.57 Gb of Illumina clean data, 52.95 Gb of Hi-C clean data, and 14.95 Gb of RNA-seq data were obtained. The final assembled genome size was 195.21 Mb with a scaffold N50 of 49.30 Mb, and 97.24% of the sequences were successfully anchored to 4 chromosomes. Annotation identified 10,909 protein-coding genes (91.48% of which were functionally annotatable), along with 73 rRNAs, 92 small RNAs, 82 regulatory RNAs, 374 tRNAs, 11,870 simple sequence repeats, 6053 tandem repeats, and 478,622 transposable elements. Phylogenetic analysis revealed that *Ph. chinensis* is phylogenetically closest to *Ph. papatasi*, with an estimated divergence time of approximately 27.1 million years ago. Gene family evolution was dominated by contraction, with 229 expanded and 575 contracted gene families identified in the *Ph. chinensis* branch. Additionally, 209 positively selected genes were detected, which are crucial for immune response regulation and metabolic processes related to its vectorial capacity. Furthermore, 95 P450 genes were identified, classified into four subfamilies: CYP2, CYP3, mitochondrial CYP (mito), and CYP4.

**Conclusions:**

A high-quality chromosome-level genome assembly of *Ph. chinensis* is reported here for the first time. This assembly serves as a critical genomic resource to advance research into the vector biology, insecticide resistance mechanisms, and evolutionary history, and lays a solid foundation for the development of precision VL control strategies in China.

**Graphical Abstract:**

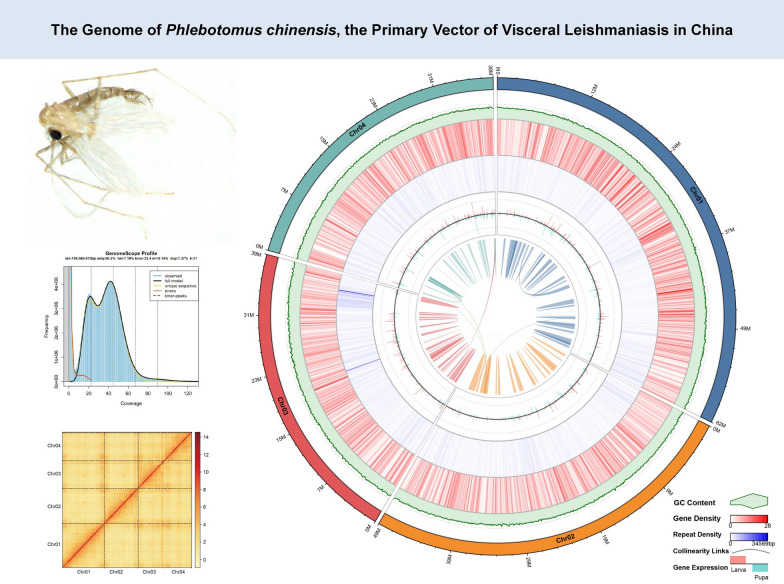

**Supplementary Information:**

The online version contains supplementary material available at 10.1186/s40249-026-01417-w.

## Background

Sandflies are blood-sucking arthropods classified within the class Insecta, order Diptera, and they play a significant role as medical vectors [1]. These insects are capable of transmitting various pathogens, including *Leishmania* [2], *Bartonella* [3], phleboviruses [4], and other virus [5] which pose substantial threats to human health. Historically, leishmaniasis was widespread in China, predominantly manifesting as visceral leishmaniasis (VL) [6, 7]. Four species of the genus *Phlebotomus* are traditionally recognized as vectors for visceral leishmaniasis in China, including *Ph. chinensis*, *Ph. longiductus*, *Ph. wui*, and *Ph. alexandri* [8].

*Ph. chinensis* is the most widely distributed species in China, and it has been reported in 358 counties across 21 provinces [8]. *Ph. chinensis* exhibits a broad distribution across China, ranging from Changchun, Jilin Province (43° 90′ N, 125° 50′ E) in the north, Hekou, Yunnan Province (23° 40′ N, 104° E) in the south, and Zhangye, Gansu Province (38° 90′ N, 100° 40′ E) in the west, to Jilin, Jilin Province (43° 80′ N, 126° 60′ E) in the east. *Ph. chinensis* is the most predominant species in plain, mountainous, and Loess Plateau regions within the area ranging from 32° to 43° N and 102° to 121° E, which is parallel to the geographical distribution of VL in China [8].

Following a nationwide campaign aimed at preventing and controlling this disease, the incidence of leishmaniasis in China has significantly decreased. As of the second half of 1958, the prevalence of VL had been controlled in the vast majority of regions in China, with only a few cases being reported in the western areas [9]. However, in recent years, there has been a notable resurgence of VL cases in the northwest region of China, particularly in Henan, Shanxi and Gansu provinces [10–12]. This recurrence has drawn significant attention from Chinese disease prevention and control institutions. Therefore, it is urgent to strengthen the monitoring of VL and phlebotomine sandflies, and deepen relevant research work to provide scientific support for epidemic prevention and control [9].

Whole-genome sequencing (WGS) is a comprehensive approach for analyzing entire genomes, offering a wealth of information that serves as the foundation for investigating critical molecular functions and gaining an in-depth understanding of biological behavior [13]. While the genome of *Drosophila melanogaster* [14], a widely used insect model organism, as well as those of several dipteran species such as *Anopheles gambiae* [15], *An. sinensis* [16], *Aedes aegypti* [17], and *Ae. albopictus* [18, 19] have been reported, progress in sequencing the genomes of sand flies has remained relatively limited. Recently, the genomes of *Ph. papatasi* and *Lutzomyia longipalpis* have been published [20, 21].

However, *Ph. chinensis* differs from *Ph. papatasi* and *Lu. longipalpis* in terms of its distribution areas and its capacity to transmit pathogens, suggesting the importance of sequencing the entire genome of *Ph. chinensis*. The absence of a stable laboratory strain, coupled with the high level of genetic variability among field-collected sand flies, poses a significant challenge to obtaining high-quality genomic data for this species. To address this critical gap, the first chromosome-level genome assembly of *Ph. chinensis* was generated in this study.

## Methods

### Sample collection

Due to the absence of laboratory strains of *Ph. chinensis*, a special breeding site was selected for sample collection. Sandfly specimens for this study were obtained from a chicken coop located in Hedi Village, Yangquan City, Shanxi Province, China. This area located in the Loess Plateau extension, and the chicken coop is situated on a hillside surrounded by mountainous forests with relatively soft soil. *Ph. chinensis* constitutes the predominant population locally.

The sandflies were collected using light traps and subsequently anesthetized with chloroform the following morning. Samples were sorted under stereomicroscopy. Female and male sandflies were stored separately before being transported to the laboratory for further experimentation. The specimen of sandflies was identified based on the morphology of the cibarium, spermathecae and male genitalia, as well as cytB fragment sequencing [22, 23]. Some live sandflies were collected from the samples. Larvae and pupae were obtained following short-term rearing in the laboratory, and individual 3rd-instar larvae and pupae were selected for RNA sequencing.

### Genome sequencing

**Fig. 1:**
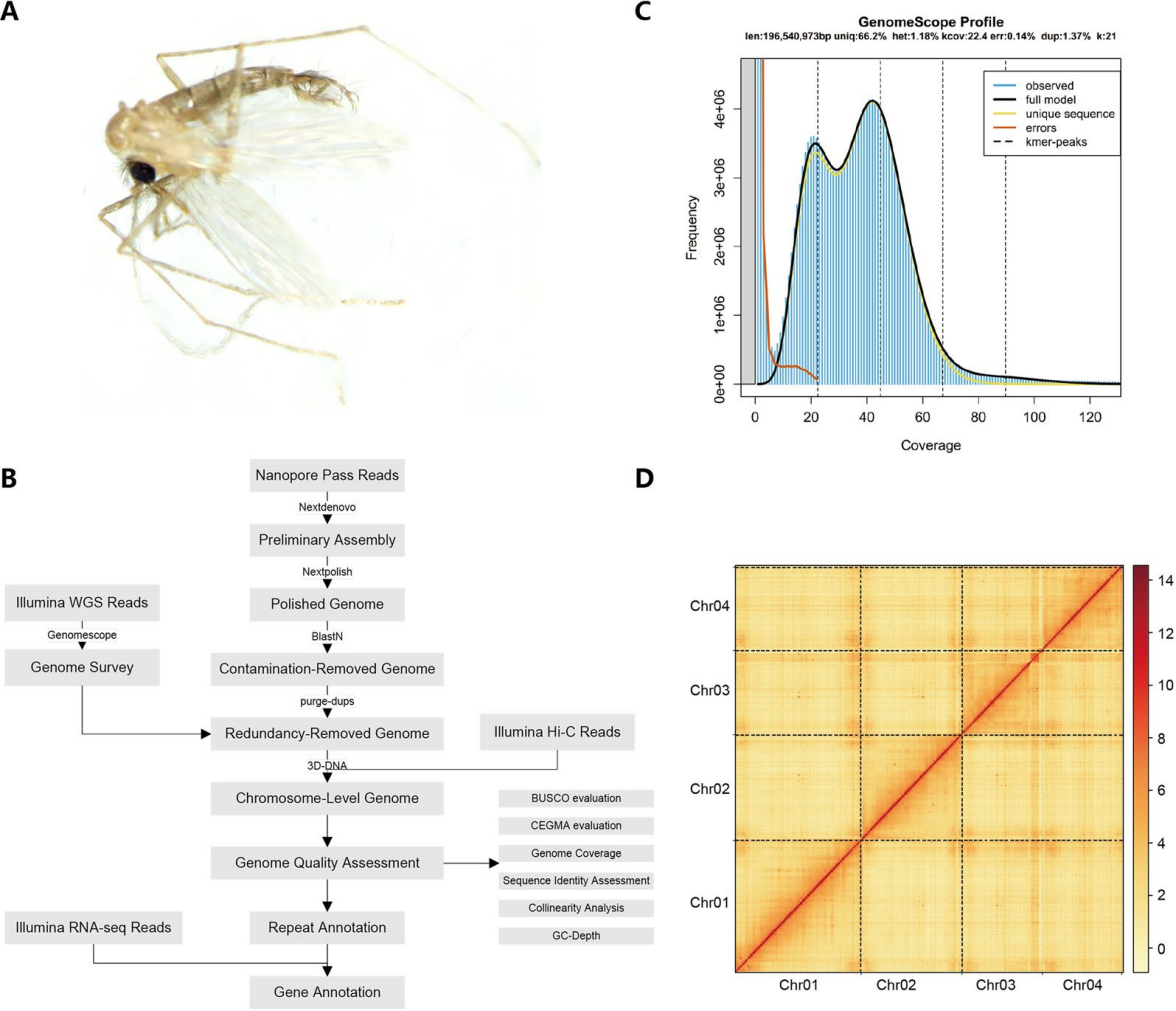
De novo assembly workflow and characteristics of the *Phlebotomus chinensis* genome. **A** Morphological photograph of an adult *Ph. chinensis*. **B** Workflow of the de novo genome assembly of *Ph. chinensis*, illustrating the complete process from sequencing data to genome assembly, contamination removal, redundancy removal, chromosome scaffolding, and gene annotation. **C** 21 K-mer distribution plot analyzed by GenomeScope, showing the coverage distribution of observed sequences, full k-mer model, error sequences, and unique sequences, as well as k-mer peaks, which are used to assess genome size, heterozygosity, and other characteristics. **D** Hi-C interaction heatmap for chromosome scaffolding, showing the interaction intensity between four chromosomes (Chr 01–Chr 04) of *Ph. chinensis*. The darker the color, the stronger the interaction

The third-generation sequencing technology Nanopore and the next-generation platform Illumina were employed in this study. The data obtained from third-generation sequencing served as the benchmark, while both second- and third-generation sequencing data were utilized for correction purposes. The sequencing strategy and analysis pipeline are illustrated in Fig. 1B.

#### Genome extraction and quality assessment

Individual male *Ph. chinensis* was used for DNA extraction and subsequent genomic survey sequencing (Fig. 1A). Based on the total amount of DNA extracted from a single individual, the yield of purified DNA, and the required library construction starting amount for third-generation sequencing, we ultimately selected fifty male *Ph. chinensis* for DNA extraction and subsequent Oxford Nanopore Technologies (ONT) sequencing. Fifty male sandflies were utilized as a pool for genomic DNA extraction, chosen to avoid contamination from host blood meals in females. Genomic DNA was extracted using the QIAGEN® Genomic Kit (QIAGEN, Hilden, Germany), following the standard procedures outlined in the kit's instructions. The quality of the genomic DNA was assessed through agarose gel electrophoresis, spectrophotometry, and Qubit assays. Only samples that met quality standards were employed for high-throughput sequencing library construction as described below.

#### Illumina sequencing

For Illumina sequencing, high-throughput sequencing libraries were generated using the Truseq Nano DNA HT Sample Preparation Kit (Illumina, San Diego, USA) in accordance with the manufacturer's recommendations. The constructed libraries were sequenced on the Illumina NovaSeq platform, yielding 150 bp paired-end reads with an insert size of approximately 350 bp. Raw data filtering was performed using fastp v0.21.0, followed by quality statistics assessment of the clean data [24].

#### Nanopore sequencing

A total of 2 µg of DNA per sample was utilized for the ONT library preparations. Long DNA fragment size selection was performed using the BluePippin system (Sage Science, Beverly, MA, USA), with the retention of only those fragments longer than 10 kb. Subsequently, the ends of the DNA fragments were repaired, and an A-ligation reaction was conducted with the NEBNext Ultra II End Repair/dA-tailing Kit (NEB, Ipswich, UK). The adapter from the LSK109 kit was employed for further ligation reactions, and a Qubit® 3.0 Fluorometer (Invitrogen, Carlsbad, USA) was used to quantify the size of library fragments. Sequencing was then carried out on a Nanopore PromethION sequencer (Oxford Nanopore Technologies, Oxford, UK) at Grandomics Biosciences in Wuhan, China [25]. Nanopore sequencers output FAST5 files containing signal data. Basecalling was initially performed to convert these FAST5 files into FASTQ format using Guppy Version 3.2.2 [26]. The raw reads in FASTQ format with mean_qscore_template < 7 were subsequently filtered to yield passing reads.

#### HiC sequencing

To anchor hybrid scaffolds onto the chromosome, genomic DNA was extracted from *Ph. chinensis* to construct the Hi-C library. Then, we constructed the Hi-C library and obtained sequencing data via the Illumina NovaSeq platform. In brief, the abdomens of fifty individuals (from the same batch of specimens used for third-generation ONT sequencing) were removed and infiltrated with nuclei isolation buffer supplemented with 2% formaldehyde. Crosslinking was stopped by adding glycine followed by additional vacuum infiltration. Fixed tissue was then ground to powder before being re-suspending in nuclei isolation buffer to prepare a nuclear suspension. The purified nuclei were digested with 100 units of DpnII and marked by incubating with biotin-14-dCTP. Biotin-14-dCTP from non-ligated DNA ends was removed using to the exonuclease activity of T4 DNA polymerase. The ligated DNA was sheared into 300 − 600 bp fragments, and then blunt-end repaired and A-tailed, followed by purification through biotin-streptavidin-mediated pull down. Finally, the Hi-C libraries were quantified and sequenced using the Illumina NovaSeq platform.

#### RNA-seq

Individual 3rd-instar larvae and pupae were used for RNA-seq. For the Illumina-based sequencing workflow, total RNA was first extracted from each sample using TRIzol reagent (Invitrogen, Carlsbad, USA). Samples were homogenized in TRIzol reagent, mixed with chloroform, and centrifuged. The aqueous phase was then collected. Isopropanol-precipitated RNA was washed with 75% (v/v) ethanol, dried, and resuspended in RNase-free water. RNA quality was verified via the NanoDrop and the Agilent 2100 Bioanalyzer. Subsequently, cDNA libraries were constructed using the Illumina TruSeq Stranded mRNA Library Prep Kit. mRNA was enriched, fragmented, and reverse-transcribed into cDNA. After end repair, A-tailing, and adapter ligation, 300–400 bp fragments were selected and PCR-amplified. Libraries were quantified by quantitative real-time PCR (qRT-PCR) and qualified via the Agilent 2100 Bioanalyzer. Finally, qualified libraries (2 nmol concentration) were pooled, denatured, and sequenced on the Illumina NovaSeq 6000 platform in paired-end 150 bp (PE150) sequencing mode.

### Genome assembly and quality assessment.

#### Genome survey

For genome survey, raw illumina short reads were first quality-controlled using fastp v0.21.0 (-n 0, -f 5, -F 5, -t 5, -T 5). High-quality clean data were obtained by removing adapter sequences, low-quality reads (Phred score < 20) and reads with any N bases, as well as trimming 5 bases from both the 5' and 3' ends of both reads. Clean data were used to generate 21-mer frequency distribution via Jellyfish v2.3.0, a key to estimating genome characteristics. Two tools analyzed the 21-mer spectrum for genome size and heterozygosity. GenomeScope v1.0.0 used a Bayesian framework to model k-mer distribution for estimation. GCE v1.0.2 calculated genome size via distinct k-mer number and average coverage, and heterozygosity via heterozygous-to-total k-mer ratio.

#### Genome assembly

For de novo genome assembly, an ONT-only assembly was constructed utilizing an overlap-layout-consensus (OLC)/string graph method with NextDenovo v2.3.1. The original subreads were initially self-corrected using NextCorrect v2.3.1 to obtain consistent sequences [consistent sequence (CNS) reads]. The preliminary genome assembly was performed based on the correlation of CNS reads as processed by the NextGraph module v2.3.1. To enhance the accuracy of the assembly, contigs were refined using Racon v1.3.1 with ONT long reads and subsequently polished with Illumina short reads through NextPolish v1.3.0 [27]. Considering the impacts of species heterozygosity and multi-individual pooling, purge_dups v1.2.6 (-x map-ont -t 40 -2 -T cutoffs -c) was used to perform de-redundancy on the corrected genome.

#### Genome quality assessment

The completeness of the genome assembly was evaluated using BUSCO v5.1.3 [28] (Benchmarking Universal Single Copy Orthologs) with the insecta_odb10 database as the reference dataset and CEGMA v2 [29] (Core Eukaryotic Gene Mapping Approach). To assess the accuracy of the assembly, all Illumina paired-end reads were mapped to the assembled genome utilizing BWA 0.7.12-r1039 (Burrows-Wheeler Aligner) [30]. The mapping rate and genome coverage of the sequencing reads were subsequently analyzed using SAMtools v1.4 [31]. Additionally, base accuracy of the assembly was determined using BCFtools v1.8.0 [32]. The third-generation sequencing reads were aligned to the genome using Minimap2 [33], and various metrics such as alignment rate, depth, and GC content of the reads were calculated.

#### Assessment of exogenous genomic contamination

The genome sequences were segmented, with sequences of length ≤ 1 Mb remaining unsegmented, while sequences exceeding 1 Mb were divided into segments of 50 kb per bin to create a new genome sequence file. The tool BLASTn v2.9.0 was employed to align the segmented genomes against the NT Sequence Database. The optimal alignment result for unsegmented sequences was deemed the final outcome. For those sequences that had been segmented into bins, the species with the highest number of alignments was considered the final result. Additionally, results from sequence alignment to each species were compiled to evaluate potential exogenous genomic contamination.

### Genome Hi-C assisted assembly

Prior to chromosome-level assembly, an index was built for the contig-level assembled genome. Hi-C raw data were filtered using fastp v0.21.0, and the cleaned data were aligned with Juicer v2.20 to construct a standardized interaction matrix. Chromosome assembly and scaffolding were then performed using 3D-DNA v180114, which executes automatic multi-round iterative optimization, verifies assembly accuracy based on inter-scaffold interaction strength, and eliminates abnormally linked fragments. For potential residual issues including scaffold misassembly, orientation reversal, and local breaks, Juicebox v1.11.08 was employed for visualized manual correction. Ultimately, plotHicGenome was used to generate chromosome-wide Hi-C interaction heatmaps.

### Genome annotation

#### Repeat annotation

##### Tandem repeat analysis

Tandem repeats (TR) encompass various types of DNA, including Satellite DNA, Minisatellites, and Microsatellites. In this study, GMATA v2.2 [34] and TRF (Tandem Repeats Finder v4.07b) [35] were employed to conduct a genome-wide search for tandem repeats using default parameters. GMATA specifically targets microsatellite sequences, while TRF is designed to identify all forms of tandem repeats.

##### Analysis of transposable elements

Dispersed repeats, commonly referred to as transposable elements (TEs), were investigated using a systematic approach. Initially, MITE-Hunter was employed to identify MITE small transposons within the genome [36], resulting in the creation of a MITE library file (TE.lib). The genome underwent hard masking once, with repeat sequences designated as 'N'. Subsequently, these repeat sequences were subjected to de novo identification utilizing RepeatModeler 1.0.11 [37], leading to the formation of a de novo library file (RepMod.lib). Finally, the TE.lib, RepMod.lib, and Repbase libraries were integrated to generate a comprehensive total library file. The obtained library was then aligned to TEclass Repbase (http://www.girinst.org/repbase) to classify the type of each repeat family. For further identification of the repeats throughout the genome, RepeatMasker 1.331 [37] was applied to search for known and novel TEs by mapping sequences against the de novo repeat library and Repbase TE library. Overlapping transposable elements belonging to the same repeat class were collated and merged.

#### Gene structure annotation

Three methods were employed for gene structure prediction: transcriptome prediction, homologous protein prediction, and de novo prediction. The PASA (Program to Assemble Spliced Alignments) v2.3.3 [38] was utilized to conduct gene predictions based on the transcriptome data (The transcriptome dataset can be found on the GenBank website with the access number PRJNA1199781 or the NGDC website with the number PRJCA042299). Homology-based protein predictions were carried out using GeMoMa v1.6.1 [39], leveraging the protein sequence information from closely related species, including *Ph. papatasi*, *Lu. longipalpis*, *Dr. melanogaster*, *An. funestus*, *Culex pipiens*, and *Ae. albopictus*. For de novo prediction, 3000 reliable genes were selected from those identified through transcriptome analysis and these genes were subsequently used to train AUGUSTUS v3.3.1 [40] to develop a species-specific predictive model. This training model facilitated de novo gene structure predictions using AUGUSTUS v3.3.1. Ultimately, an initial genome gene set comprising predicted genes was generated by integrating results from EvidenceModeler v1.1.1 [38]. To refine this initial set further, TransposonPSI [41] alignment was employed to remove genes containing transposable elements as well as those with coding errors; thus yielding the final gene set.

#### ncRNA annotation

Non-coding RNA (ncRNA) encompasses various types, including ribosomal RNA (rRNA), transfer RNA (tRNA), small nuclear RNA (snRNA), small nucleolar RNA (snoRNA), and microRNA (miRNA). The cmscan tool within the Infernal program v1.1.2 [42] was employed to compare against the Rfam database [43] for the prediction of genomic ncRNAs. tRNAscan-SE v2.0 [44] was utilized specifically for tRNA prediction, while RNAmmer v1.2 [45] was applied to construct a model for rRNA and its subunit prediction.

#### Gene function annotation

According to the predicted protein sequence of the gene, five databases were utilized to annotate gene functions: NR (Non-Redundant Protein Sequence Database, https://www.ncbi.nlm.nih.gov/protein), KEGG (Kyoto Encyclopedia of Genes and Genomes, https://www.kegg.jp/) [46], KOG (Eukaryotic Orthologous Groups, https://www.ncbi.nlm.nih.gov/research/cog-project/) [47], GO (Gene Ontology, https://geneontology.org/) [48], and the SwissProt database (https://www.sib.swiss/swiss-prot). The KEGG database also provided annotations regarding the biological pathways in which these genes may be involved. In the GO database, genes were classified and annotated based on their Biological Process (BP), Cellular Component (CC), and Molecular Function (MF). The annotation results from NR, KEGG, KOG, GO, and SwissProt databases were compared for overlap. Ultimately, the union of all annotation results was used as the final comprehensive annotation outcome.

### Genome evolution analysis

#### Identification of orthologous and paralogous gene families

OrthoFinder v3.1.1 was employed to identify shared orthologs among *Ph. chinensis*, *Ph. papatasi* (GCF_024763615.1), *Lu. longipalpis* (GCF_024334085.1) and *Ae. albopictus* (GCF_035046485.1), *An. funestus* (GCF_943734845.2), *Cu. pipiens* (GCF_016801865.2) and *Dr. melanogaster* (GCF_000001215.4). Relevant genomic sequences and annotation files were retrieved from the NCBI database. For each species, alternative splicing variants of protein-coding genes were removed, and the longest transcript was retained, while genes containing internal stop codons were filtered out.

OrthoFinder first performed an all-vs-all sequence alignment of all protein sequences [49, 50]. Subsequently, the MCL algorithm was utilized to cluster genes into orthologous gene families, paralogous gene families, and single-copy orthologs. From the gene family clustering results, genes categorized as "unique genes" or "unclustered genes" were defined as species-specific genes, and GO and KEGG enrichment analyses were performed using ClusterProfiler v4.14.4 [51].

#### Phylogenetic tree

Single-copy orthologous genes were employed for phylogenetic tree inference and divergence time estimation. Protein sequence multiple sequence alignment (protein-MSA) was conducted using MAFFT [52], followed by the removal of low-quality and high-gap regions via trimAl (v1.5) [53]. Subsequently, the alignment results of each SCO were concatenated into a partitioned supermatrix with partition information by gene partitioning. Finally, a maximum-likelihood (ML) species tree was constructed using IQ-TREE (v3.0.1), with model selection and parameter estimation performed by integrating the MFP (ModelFinder Plus) strategy of ModelFinder [54].The species tree was rooted using *Dr. melanogaster* as the outgroup.

#### Divergence time

For molecular dating, we specified node-age calibrations (e.g., fossil constraints) as temporal priors on the rooted topology. Using IQ-TREE 3 (–dating mcmctree), we computed the gradient vector(s) and Hessian matrix/matrices of branch lengths at their maximum-likelihood estimates (for approximate-likelihood dating) and generated the corresponding MCMCtree input files. We then performed Bayesian divergence-time estimation in the approximate-likelihood framework implemented in MCMCtree (v4.10.8.iq2mc) under the EQUAL (global strict-clock) model, which assumes a single substitution rate shared across all branches. Posterior samples yielded an ultrametric, time-calibrated phylogeny with 95% credible (HPD) intervals for node ages. [55]. The fossil calibrations were based on the species pairs (*Dr. melanogaster* vs. *Lu. Longipalpis*) and (*Ae. albopictus* vs. *Cx. pipiens*).

#### Gene family expansion and contraction analysis

Gene family expansion and contraction analysis was performed using CAFE v4.2.1 [56]. The input data included a gene family count matrix file (derived from the previous gene family clustering results) and an ultrametric tree (obtained from the divergence time estimation results of MCMCtree described earlier), thereby yielding the dynamics of gene family expansion and contraction across different evolutionary branches for each studied species. For gene families with significant expansion or contraction in each species, GO functional enrichment analysis and KEGG pathway enrichment analysis were conducted using ClusterProfiler v4.14.4 [51].

#### Genes under positive selection

Single-copy orthologous genes were used as input data, and selection pressure parameters (ω, i.e., the dN/dS ratio) on the *Ph. chinensis* branch were calculated via the codeml module of PAML using the branch-site model [55]. The statistical significance of selection acting on each gene was tested with the chi-square test (*P* ≤ 0.05), and positively selected genes specific to the *Ph. chinensis* branch were ultimately identified through screening. Subsequently, GO functional enrichment analysis and KEGG pathway enrichment analysis were performed on these positively selected genes using ClusterProfiler v4.14.4 [51].

#### Individually focused gene families

Precise manual annotation was performed for the targeted gene families. Taking the P450 family as an example, the protein sequence files (pep files) of *Ph. chinensis*, *Lu. longipalpis*, and *Ph. papatasi* were subjected to Blastp v2.7.1 alignment against 2942 manually curated CYP sequences from a wide range of arthropods, followed by screening using HMM models. Abnormal sequences with premature stop codons were excluded from the alignment results. Identification results for the P450 family indicated that HMM models could effectively cover most member genes of this family, and all remaining targeted gene families were identified via HMM models. For phylogenetic analysis of the P450 family, additional partial homologous sequences of *An. gambiae* were included. Following protein sequence alignment with MAFFT v7.313, a phylogenetic tree was constructed in RAxML under the GTRGAMMA substitution model with 1000 bootstrap replicates, with human P450 sequences serving as the outgroup. For the remaining targeted gene families, phylogenetic analysis involved protein sequence alignment with MAFFT v7.313, followed by the construction of neighbor-joining (NJ) trees using IQ-TREE v3.0.1.

Software used in this study, along with their versions and parameters were summarized in Table S23.

## Results

The genomic dataset has been deposited in GenBank (https://www.ncbi.nlm.nih.gov/genbank/) under accession number PRJNA1233448 and in the National Genomics Data Center (NGDC, https://ngdc.cncb.ac.cn/) under accession number PRJCA042135.

### Genome sequencing

In this study, the complete genome of *Ph. chinensis* was sequenced using a combination of Nanopore third-generation sequencing and Illumina next-generation sequencing (Table 1S). For the Nanopore sequencing, the total data generated amounted to 127.05 Gb, with total pass reads number of 7,967,112. The pass reads mean length was 15.9 Kb, while the Pass reads N50 length was recorded at 23.2 Kb. The pass reads maximum length reached 764.3 Kb. In terms of Illumina sequencing, the total clean data obtained was 10.57 Gb. For Hi-C sequencing, a total of 353,539,722 clean reads were generated, corresponding to 52.95 Gb of clean bases with a Q30 value of 92.94%. For RNA-seq, two datasets were obtained from larval and pupal samples. The larval sample yielded 56,638,814 clean reads (8.56 Gb clean bases) and a Q30 value of 93.64%, while the pupal sample generated 42,426,172 clean reads (6.39 Gb clean bases) with a Q30 value of 94.91% (Table S1).

### Genome assembly and quality assessment

Genome survey results indicated that the estimated genome size of this species ranges from 196 to 208 Mb, with a heterozygosity of approximately 1.0% to 1.2% (Fig. 1C, Table S2). Following de novo assembly and multiple rounds of data polishing, the preliminary genome assembly was determined to be 230.79 Mb in size, with a contig N50 of 0.94 Mb and 707 contigs (Table S3). Subsequent quality control analyses identified 8 contaminated contigs, all of bacterial origin. Affter removing these contaminated sequences and redundant regions, the final genome assembly was finalized at 194.79 Mb, with a contig N50 improved to 1.31 Mb and 453 contigs remaining (Table S4).

Quality assessment results showed a BUSCO completeness of 97.7%, including 96.7% complete and single-copy BUSCOs, 1.0% complete and duplicated BUSCOs, 0.6% fragmented BUSCOs, and 1.8% missing BUSCOs (Table S5). CEGMA analysis revealed that 237 out of 248 core genes (95.56%) were successfully assembled, with 218 (87.90%) classified as complete core genes. Mapping rates of ONT and Illumina data to the assembled genome were 97.90% and 99.74%, respectively. Based on Illumina mapping results, 4765 homozygous SNPs and 2268 homozygous Indels were identified, corresponding to a single-base accuracy of Q40 (Table S6). Sequencing coverage analysis demonstrated an average genomic coverage depth of 454.95×, with 99.97% of the genome covered at ≥ 1 × depth. The GC content of the genome ranged from 30 to 50%, and sequencing depths were predominantly concentrated between 400× and 700×.

### Genome chromosome Hi-C assists assembly

A total of 176,769,861 pairs of clean reads were subjected to Hi-C analysis. Only 2.46% of the reads failed to map to the reference genome, 74.24% contained ligation motifs. 43.57% ultimately formed valid Hi-C interactions, corresponding to an approximate sequencing depth of 118.36×. Of these valid interactions, interchromosomal interactions accounted for 24.50%, intrachromosomal interactions for 19.06%, and long-range (> 20 kb) interactions for 6.99% (Table S7). Among short-range interactions, those < 500 bp had the highest proportion, which was consistent with the typical distance distribution characteristics of Hi-C data.

After 3D-DNA clustering and manual curation, a total sequence length of 189.83 Mb was mapped to four chromosomes (Fig. 1D), representing 97.24% of the total length of the final genome assembly. Within each chromosome cluster, contigs were organized and all orientations of contigs were traversed using a weighted directed acyclic graph (WDAG). Ultimately, contigs with confirmed order and orientation were incorporated to generate the final chromosomal-level genome sequence by appending an additional 500 Ns. This assembly had a size of 195.21 Mb, with a contig N50 of 1.31 Mb and a scaffold N50 of 49.30 Mb. A further BUSCO assessment was performed on the Hi-C scaffolding results, which yielded a completeness of 97.7%, consistent with that of the pre-scaffolding assembly.

**Fig. 2:**
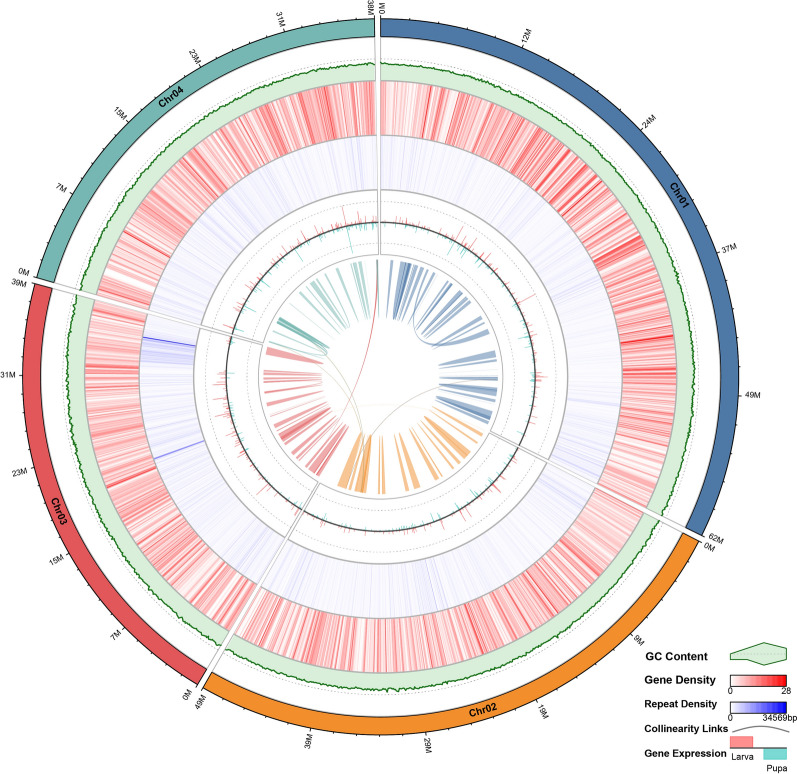
Genome feature circular plot of *Phlebotomus chinensis*. The genome feature circular plot of *Ph. chinensis* consists of six concentric circles. The outermost circle displays the four chromosomes along with their lengths. Moving inward, the second circle uses the height of green polygons to depict the GC content across the genome. The third circle illustrates gene density through the intensity of red hues, with darker red indicating a higher density of genes. The fourth circle represents repeat sequence density via the intensity of blue hues, where darker blue signifies a higher density of repeat sequences, and circles 2–4 are all calculated with a window size of 10 kb. The fifth circle shows gene expression, with the heights of pink (Larva) and turquoise (Pupa) bars (in two orientations) indicating gene expression levels at different developmental stages. The innermost circle presents collinearity links, with collinear regions within the same chromosome are denoted by links of a single color

For the Hi-C-based chromosome-level genome assembly, the number of Hi-C Read Pairs between any two bins was utilized as an indicator of interaction intensity between those bins. Four distinct chromosome groups were clearly identified, which ass consistent with the chromosome numbers of *Ph. papatasi* and *Lu. longipalpis* [57]. Within each group, the strength of interactions at diagonal positions exceeded that at off-diagonal positions, and there was minimal noise outside the diagonal region, indicating a high-quality genome assembly (Fig. 2). Chromosomes 01, 02, 03, and 04 contained 109, 80, 184, and 93 contigs, respectively (Table S8). However, since karyotype analysis was not performed in the present study, further experimental validation would be required in the future.

### Genome annotation

Genome assembly and annotation datasets of *Ph. chinensis* have been uploaded to 10.5281/zenodo.18193263.

#### Repeat sequence prediction

##### Tandem repeat analysis

GMATA software analysis revealed the presence of 11,870 SSR sequences in the genome of *Ph. chinensis*, with a cumulative length of 141,198 bp, which constitutes approximately 0.07% of the total genome length. Among these, there were 10,646 two-nucleotide repeat units totaling 121,238 bp (85.86%), and 1132 three-nucleotide repeats amounting to 18,108 bp (12.82%). Additionally, there were 92 four-nucleotide repeats with a combined length of 1852 bp (1.31%)(Table S9).

The TRF software was employed to analyze the tandem repeats present in the genome using default parameters. The analysis revealed a total of 6053 tandem repeats, with an aggregate length of 1,080,321 bp, which constitutes approximately 0.55% of the overall genome length.

##### Analysis of transposable elements (TEs)

Utilizing the software RepeatMasker v1.331 (https://www.repeatmasker.org) for TE prediction, a total of 359,617 TEs within the *Ph. chinensis* genome were revealed, including 70,871 long terminal repeats (LTRs), 51,600 long interspersed nuclear elements (LINEs), 6892 short interspersed nuclear elements (SINEs), 15,961 rolling-circle elements (RCs), and 8788 miniature inverted-repeat transposable elements (MITEs) (Figure S1A, Table S10).

#### Coding gene prediction

Coding gene prediction relied on transcriptome analysis, homologous protein predictions, and de novo approaches. A total of 15,430 genes were predicted based on transcriptomic data using PASA v2.3.3. Additionally, GeMoMa v1.6.1 was employed to predict 14,923 genes utilizing the protein sequence information from closely related species. From the genes identified through transcriptome analysis, 3000 reliable candidates were selected to train AUGUSTUS to develop a gene prediction model for *Ph. chinensis* aimed at de novo gene prediction. Ultimately, a total of 12,598 genes were predicted through this de novo approach.

Based on the aforementioned three prediction results, the Evidence Modeler (EVM) was employed to integrate these predictions according to a specified weight value (PASA ≥ GeMoMa ≥ de novo prediction), resulting in a predicted gene set for *Ph. chinensis*. Subsequently, using TransposonPSI alignment, genes containing transposable elements and coding errors were excluded from the initial predicted gene set, leading to the final gene set. Ultimately, a total of 10,909 coding genes were predicted within the *Ph. chinensis* genome. The average length of these genes was found to be 10,452.29 bp, with an average CDS length of 1585.82 bp. Furthermore, the average number of exons in each gene was 4.54; the average exon length was recorded at 397.26 bp while the average intron length measured 1652.61 bp.

#### Non-coding RNA (ncRNA) prediction

The software Infernal v1.1.2 was utilized to compare the genome sequences with the Rfam database for the prediction of genomic non-coding RNAs (ncRNAs). tRNAscan-SE v2.0 was employed to predict tRNAs, while RNAmmer v1.2 was applied to construct a model for predicting rRNA and its various subunits. Ultimately, a total of 73 rRNAs, 92 small RNAs, 82 regulatory RNAs, and 374 tRNAs were predicted (Table S11).

#### Gene function annotation

Genes were annotated in five functional databases with the following counts: NR (9885), KEGG (6558), KOG (7583), GO (6680), and SwissProt (8711) (Table S12). In total, the number of genes that could be annotated to functional databases reached 9980, which accounted for 91.48% of the coding genes. The Venn diagram illustrating the overlap of annotated genes was presented in Figure S1B.

#### Genome comparison with *Ph. papatasi* and *Lu. longipalpis*

The *Ph. chinensis* genome was compared with those of *Ph. papatasi* and *Lu. longipalpis* (Table 1, Figure S2). The comparison revealed that the total number of genes, average CDS length, average number of exons per gene and average exon length were similar across the three sandfly species. *Ph. papatasi* exhibited the longest average transcript length and average intron length, followed by *Ph. chinensis*, while *Lu. longipalpis* had the shortest ones (Table S13).

**Table 1:** Genome quality comparison of *Ph. chinensis*, *Ph. papatasi* and *Lu. longipalpis*

	***Ph. chinensis***	***Ph. papatasi***		***Lu. longipalpis***	
		Old	New	Old	New
Genome Size	195,208,748 bp	363,767,908 bp	351,623,088 bp	154,229,266 bp	147,838,017 bp
Depth	454.95 x	15.1 x	113.5 x	38.9 x	53 x
Contig N50	1.31 MB	5.8kb	926.6 kb	7.5kb	1,092.5 kb
Contig Count	453	139,199	1,349	35,969	255
Scaffold N50	49.30 Mbp	27,956 bp	111.8 Mbp	85,093 bp	40.6 Mbp
Scaffold Count	145	106,826	645	11, 532	4
Coding Genes	10,909	11,377	11,610	10,422	11,236
Noncoding Genes	621	444	995	338	778
BUSCO	97.70%	86.50%	95.20%	86.10%	96.60%
NCBI Accession	Not registered	GCA_000262795. 1	GCF_024763615. 1	GCA_000265325. 1	GCF_024334085. 1
VectorBase	Unregistered	Past	Current Reference	Past	Current Reference

### Evolution analysis

#### Identification of orthologous and paralogous gene families

**Fig. 3:**
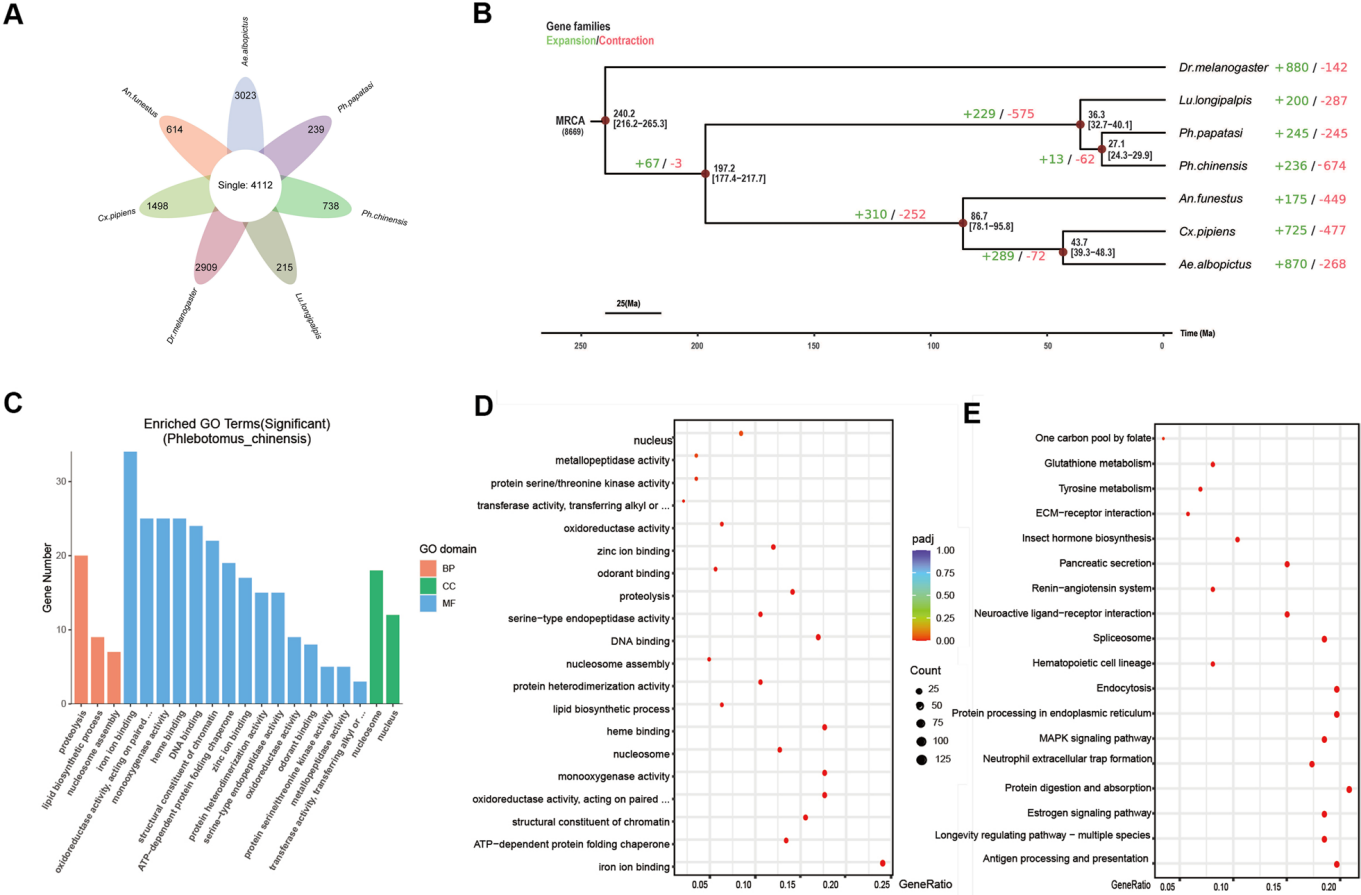
Evolution and functional enrichment analysis of expanded genes in *Phlebotomus chinensis*. **A** Petal plot for gene family identification, showing the number of shared single-copy orthologous genes and species-specific genes among *Ph. chinensis* and other related species. The central indicates the number of single-copy orthologous genes shared by all species, while the outer sides show the numbers of species-specific genes. **B** Phylogenetic tree of species combined with divergence time and gene family expansion/contraction results. Fossil times are labeled at the bottom. The numbers on the branches represent the number of expanded and contracted gene families, respectively. Pie charts on the branches use different colors to indicate expanded (e.g., green), contracted (e.g., red), and conserved (e.g., blue) gene families. MRCA denotes the most recent common ancestor, with the number of gene families in MRCA being 14,445. **C** Bar plot of GO enrichment analysis for expanded genes in *Ph. chinensis*. Three colors represent three core functional categories: biological process (BP, red), cellular component (CC, green), and molecular function (MF, blue). The x-axis shows enriched GO terms, and the y-axis shows the number of genes. **D** Bubble plot of BP enrichment results for expanded genes in *Ph. chinensis*. The x-axis is GeneRatio, the y-axis lists enriched BP terms. The size of the bubbles indicates the number of genes, and the color indicates the significance (adjusted *P*-value). **E** Bubble plot of KEGG enrichment results for expanded genes in Ph. chinensis. The x-axis is GeneRatio, the y-axis lists enriched KEGG pathways. The size of the bubbles indicates the number of genes, and the color indicates the significance (adjusted *P*-value)

A total of 4112 single-copy orthologous genes were identified among the seven species (Fig. 3A, Figure S1C). For *Ph. chinensis*, 8610 gene families were obtained through clustering, with an average of 1.26 genes per family. Among these, 35 gene families were specific to sandflies, corresponding to 118 genes, and 620 genes remained unclustered (Table S14). Sandfly-specific genes were defined as those obtained by merging the genes from sandfly-specific gene families and the unclustered genes.

Enrichment analysis showed that the 738 genes from sandfly-specific gene families were were significantly enriched to 221 GO terms (*P* < 0.05, Table S15), and 104 KEGG pathways, with 10 significantly enriched (*P* < 0.05, Table S16).

#### Phylogenetic relationships

A time-calibrated phylogenetic tree, constructed from the concatenated sequence of 4122 orthologous protein-coding genes (with *Dr. melanogaster* as the outgroup), revealed that sand flies (including *Ph. chinensis, Ph. papatasi, and Lu. longipalpis*) form a monophyletic sister group with mosquitoes, rather than clustering with Muscomorpha (Fig. 3B). This topological structure is consistent with previous research reports (citation). *Ph. chinensis* is more closely related to *Ph. papatasi*, with a divergence time of approximately 27.1 million years ago (Ma) (95% HPD: 24.3–29.9 Ma), while its divergence time from *Lu. longipalpis* is approximately 36.3 Ma (95% HPD: 32.7–40.1 Ma).

#### Gene family expansion and contraction analysis

Using CAFE and the ultrametric tree, the dynamics of gene family expansion and contraction across different evolutionary branches of each species were analyzed. The most recent common ancestor (MRCA) of the seven species possessed 8669 gene families. Subsequent evolution led to distinct gene family change patterns. Focusing on sandfly species, *Ph. chinensis* had 229 expanded and 575 contracted gene families, while *Ph. papatasi* showed 13 expansions and 62 contractions. *Lu. longipalpis* exhibited 236 expansions and 674 contractions. Sandfly species presented a more pronounced contraction in gene families. Among mosquitoes, except for Anopheles, the rest generally showed a gene expansion trend.

Enrichment analysis of expanded gene families yielded 34 GO terms (20 significantly enriched, *P* < 0.05, Table S17) and 36 KEGG pathways (18 significantly enriched, *P* < 0.05, Table S18) (Fig. 3C–E). Contracted gene families were annotated to 9 GO terms (all significantly enriched, *P* < 0.05) and 4 KEGG pathways (3 significantly enriched, *P* < 0.05).

#### Genes under positive selection

Based on Darwin’s theory of natural selection and the neutral evolution theory, the ratio of nonsynonymous substitution rate (Ka) to synonymous substitution rate (Ks) (Ka/Ks) is a widely used indicator to detect selection pressure on protein-coding genes. Using single-copy orthologous genes as input, the codeml module of PAML was employed to calculate the selection pressure acting on the *Ph. chinensis* branch. The statistical significance of selection for each gene was tested via chi-square test (*P* < 0.05), resulting in the identification of 209 positively selected genes in *Ph. chinensis*. GO and KEGG enrichment analyses of these positively selected genes revealed 109 GO terms (Table S19) and 155 KEGG pathways (Table S20).

#### Individually focused gene families

Precise manual annotation was conducted for the targeted gene families, including cytochrome P450 monooxygenases (P450), carboxylesterases (CCEs), glutathione S-transferases (GSTs), gustatory receptors (GRs), ionotropic receptors (IRs), olfactory receptors (ORs), Toll pathway genes (Toll), and IMD pathway genes (Table S21). For *Ph. chinensis*, *Ph. papatasi* and *Lu. longipalpis*, the gene counts of these families were at comparable levels, with *Ph. chinensis* harboring the lowest number of genes across all targeted families.

**Fig. 4:**
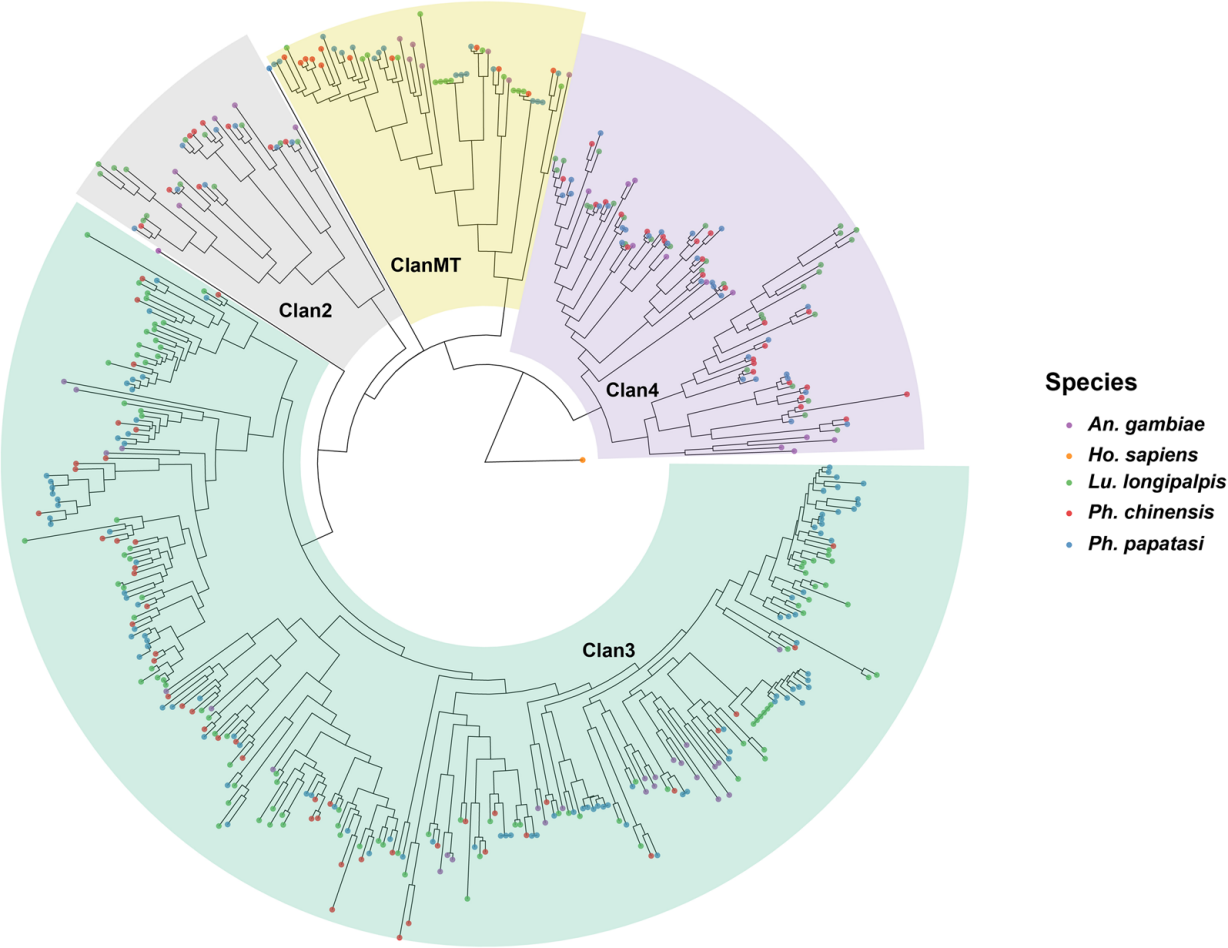
Phylogenetic analysis of p450 gene family in sandflies. This circular phylogenetic tree illustrates the P450 gene family of three sandfly species (*Ph. chinensis*, *Ph. papatasi*, *Lu. longipalpis*). *An. gambiae* P450 genes were included as references for localization, and *Ho. sapiens* serveed as the outgroup. Four colored backgrounds represent four P450 clans (Clan2, ClanMT, Clan4, and Clan3). Different colored dots correspond to different species: purple for *An. gambiae*, orange for *Ho. sapiens*, green for *Lu. longipalpis*, red for *Ph. chinensis*, and blue for *Ph. papatasi*

Taking the P450 gene family as an example, the protein sequence files (pep files) of *Ph. chinensis*, *Lu. longipalpis*, and *Ph. papatasi* were subjected to BLASTP alignment against 2942 manually curated CYP sequences from a wide range of arthropods, followed by screening with HMM models. A total of 95 P450 genes were identified in *Ph. chinensis* via the above two methods, including one additional gene detected exclusively through alignment with the 2942 curated CYP sequences (Fig. 4). Similarly, 154 and 155 P450 genes were identified in *Lu. longipalpis* and *Ph. papatasi*, respectively. Phylogenetic tree analysis classified the 95 P450 genes of *Ph. chinensis* into four subfamilies: 9 in CYP2, 48 in CYP3, 12 in mitochondrial CYP (mito), and 26 in CYP4 (Table S22).

## Discussion

This study reports the first chromosome-level genome assembly of *Ph. chinensis*, the primary vector of VL in China. The final assembly (195.21 Mb, scaffold N50 = 49.30 Mb, BUSCO = 97.7%) is of high quality, with coding regions conserved among *Ph. chinensis*, *Ph. papatasi*, and *Lu. longipalpis*, while non-coding regions (e.g., intron length) differ significantly. Phylogenetic analysis confirms *Ph. chinensis* is closest to *Ph. papatasi* (divergence ~ 27.1 Ma), with gene family evolution dominated by contraction (575 contracted vs. 229 expanded families), 209 positively selected genes (involved in immunity and metabolism), and 95 P450 genes classified into four subfamilies.

The genomes of *Ph. papatasi* and *Lu. longipalpis* were not reported until 2022 [20]. The initial assemblies had genome sizes of 363.8 Mb and 154.2 Mb for *Ph. papatas*i and *Lu. longipalpis*, respectively. However, BUSCO analysis indicated relatively low integrity (86.5% for *Ph. papatasi* and 86.1% for *Lu. longipalpis*), suggesting fragmentation and potential loss of certain genomic regions. Recently, advancements in ultra-low input long-read sequencing technology have facilitated the generation of high-quality sand fly genomes [21]. The updated assemblies of *Ph. papatasi* and *Lu. longipalpis* show significantly improved quality. Their genome sizes are 351.6 Mb and 147.8 Mb, with Contig N50 values of 926.6 kb and 1092.5 kb, and Scaffold N50 values of 111.8 Mb and 40.6 Mb, respectively. Correspondingly, the BUSCO completeness scores have increased to 95.2% for *Ph. papatasi* and 96.6% for *Lu. longipalpis*.

As the primary vector of leishmaniasis in China, *Ph. chinensis* differs from *Ph. papatasi* and *Lu. longipalpis* in terms of geographical distribution and pathogen transmission capacity. Specifically, *Ph. chinensis* is endemic to China [8], while *Ph. papatasi* has a broad geographical range extending from Morocco to the Indian coast, covering southern Europe to central and eastern Africa [58]. In contrast, *Lu. longipalpis* is exclusively distributed in the Americas [59]. Regarding pathogen transmission, *Ph. chinensis* can transmit both *L. donovani* and *L. infantum* [6], whereas *Ph. papatasi* transmits only *L. major* [60]. Although *Lu. longipalpis* has been shown to act as a permissive vector under laboratory conditions, it naturally transmits only *L. infantum* [61–64].

In terms of genomic features, Scaffold N50 of *Ph. chinensis* is shorter than *Ph. papatasi* but longer than *Lu. longipalpis*. Comparative analysis of the three sand fly species revealed comparable total gene numbers, average CDS lengths, exons per gene, and exon lengths, indicating highly conserved coding regions [20, 21]. However, notable differences existed in non-coding regions. *Ph. papatasi* had the longest average transcript and intron lengths, followed by *Ph. chinensis*, with *Lu. longipalpis* the shortest. These intron length variations may reflect evolutionary pressure-genomic characteristic interactions and could also be attributed to differences in sequencing, assembly, and annotation quality, highlighting the importance of high-quality sequencing in genomic research.

Introns play significant roles in gene regulation, including transcriptional efficiency and splicing dynamics [65]. According to population genetic theory, intron size is shaped by a dynamic balance between mutational processes and natural selection [66]. This suggests that the longer introns in *Ph. papatasi* might result from reduced selective efficiency rather than accelerated evolutionary rate. Transposable element activity could also contribute to intron length variation [67]. Additionally, intron length evolution is affected by recombination rates and selection pressures on transcriptional cost. Natural selection favors shorter introns in highly expressed genes to minimize energy expenditure during transcription, which may lead to lineage-specific length adjustments based on gene expression profiles [66, 68]. Geographic and ecological factors might further modulate these genomic patterns [69].

Phylogenetic analysis revealed that *Ph. chinensis* and *Ph. papatasi* form a sister clade, with an estimated divergence time of ~ 27.1Ma. This close evolutionary relationship is consistent with their congeneric taxonomic status, explaining the partial overlap in their biological traits (e.g., Leishmania transmission capacity). *Lu. longipalpis* diverged from *Phlebotomus* species earlier (~ 36.3 Ma), which aligns with its Americas-endemic distribution and generic-level classification, supporting the validity of the current sandfly classification system.

The dynamics of gene family evolution showed that the sandfly clade is characterized by a "contraction-dominated" pattern. Since diverging from the common ancestor of *An. funestus* and *Cx.pipiens* ~ 197.2 Ma, the sandfly clade has undergone 229 gene family expansions and 575 contractions. This widespread contraction reflects the adaptive genomic streamlining of sandflies, driven by selection pressures associated with specialized ecological niches such as hematophagy and pathogen transmission [70]. The loss of redundant genes can improve energy use efficiency and reduce genomic complexity. Contracted gene families are significantly associated with 9 GO terms and 3 KEGG pathways, mostly involving non-essential functions (e.g., non-specific metabolic pathways). The 28 expanded gene families in *Ph. chinensis* are enriched in 20 GO terms (e.g., cell adhesion, signal transduction) and 18 KEGG pathways, which may represent adaptive changes enabling *Ph. chinensis* to cope with environmental challenges and vector-specific functions. The cell adhesion-related genes may promote interactions between the midgut and Leishmania parasites, while the expansion of signal transduction pathways can enhance the ability to perceive host cues and respond to environmental stresses [70, 71].

Positive selection analysis identified 209 genes under significant selection pressure (*P* < 0.05), enriched in 109 GO terms and 155 KEGG pathways, covering key processes such as immune response, metabolic regulation, and membrane transport. This reflects the long-term coevolution between *Ph. chinensis* and pathogens (e.g., *Leishmania donovani*). Positive selection on metabolic genes may help *Ph. chinensis* adapt to the nutritional demands of hematophagy (e.g., utilization of host hemoglobin, detoxification of heme). Among specialized gene families, 95 P450 genes were identified in *Ph. chinensis*, fewer than in *Lu. longipalpis* (154) and *Ph. papatasi* (155), classified into CYP2, CYP3, CYP4, and mitochondrial CYP subfamilies involved in insecticide resistance and xenobiotic metabolism. This difference may be associated with *Ph. chinensis* inhabiting rural and mountainous areas of China with relatively low insecticide pressure. Redundant detoxification genes are lost through purifying selection, while key subfamilies (e.g., CYP3, CYP4) are retained to ensure metabolism of natural xenobiotics and low-dose insecticides.

This study identified 35 sandfly-specific gene families (118 genes) and 620 unclustered species-specific genes, enriched in GO terms related to cuticle formation, sensory perception, and reproduction, as well as KEGG pathways involved in membrane transport and signal transduction. The cuticle-related genes adapt to terrestrial and hematophagous behaviors; sensory genes enhance the ability to locate hosts and breeding sites; reproduction-related genes regulate unique life cycle traits (e.g., development in moist soil, diapause). Some specific genes may be involved in pathogen-vector interactions (e.g., mediating the attachment and survival of *Leishmania* parasites in the sandfly midgut) [71]. Their functional validation is expected to provide new targets for blocking transmission (e.g., development of transgenic sandflies, design of interaction inhibitors).

Nevertheless, the present study has several limitations. Karyotype analysis was not conducted in this study, and the chromosome number was determined based on bioinformatics analysis. Evolutionary inferences rely on a limited number of species; including more vector species (e.g., *Ph. longiductus*) could improve phylogenetic resolution. Functional annotations of expanded, contracted, and positively selected genes are mostly predictive, requiring experimental validation (e.g., RNA interference, heterologous expression). Analysis of non-coding regions (introns, transposons) is still preliminary. Future research can focus on three aspects: first, functional characterization of positively selected and sandfly-specific genes to clarify their roles in transmission, host-seeking, and environmental adaptation; second, comparative analysis of genome-wide variation among geographically distinct *Ph. chinensis* populations to explore the impact of ecological pressures on genetic diversity and vector competence; third, integrating genomic and field surveillance data to construct visceral leishmaniasis epidemic risk prediction models, supporting targeted prevention and control.

## Conclusions

Building upon previous genomic and evolutionary investigations, this study reports the first high-quality chromosome-level genome assembly of *Ph. chinensis*—the principal vector of VL in China. This genomic resource addresses a critical gap in sand fly research, offering indispensable support for exploring the vector biology, pathogen transmission mechanisms, and environmental adaptability of *Ph. chinensis*. Notably, it provides a robust scientific foundation for the precise control of VL, which has experienced a recent resurgence in northwest China. Through the integration of multiple sequencing technologies and comparative genomic analyses, this study confirms the core value of a high-quality *Ph. chinensis* genome in linking basic research to applied disease control. Furthermore, it enriches the global sand fly genomic database and delivers crucial genomic resources to mitigate the burden of leishmaniasis in China.

## Supplementary Information

Additional file 1.

Additional file 2.

Additional file 3.

Additional file 4.

## Data Availability

The datasets generated during the current study are available in the GenBank with the access number PRJNA1199781 and PRJNA1233448, or in the NGDC with the access number PRJCA042299 and PRJCA042135.

## Abbreviations

BUSCO: Benchmarking Universal Single Copy Orthologs
CCE: Carboxylesterase
CDS: Coding sequence
cDNA: Complementary DNA
CNS: Consistent sequence
CYP: Cytochrome P450
Cytb: Cytochrome b
GST: Glutathione S-transferase
GO: Gene Ontology
GR: Gustatory receptor
HPD: Highest posterior density
Hi-C: High-throughput chromosome conformation capture
IR: Ionotropic receptor
KEGG: Kyoto Encyclopedia of Genes and Genomes
KOG: Eukaryotic Orthologous Groups
LINE: Long interspersed nuclear element
LTR: Long terminal repeat
MCL: Markov cluster algorithm
MITE: Miniature inverted-repeat transposable element
miRNA: MicroRNA
MRCA: Most recent common ancestor
NCBI: National Center for Biotechnology Information
NGDC: National Genomics Data Center
NT: Non-Redundant Nucleotide Sequence Database
NR: Non-Redundant Protein Sequence Database
OLC: Overlap-layout-consensus
ONT: Oxford Nanopore Technologies
OR: Olfactory receptor
P450: Cytochrome P450 monooxygenase
PE: Paired-end
PAML: Phylogenetic analysis by Maximum Likelihood
PRJCA: Project number in National Genomics Data Center
PRJNA: Project number in GenBank
RC: Rolling-circle element
rRNA: Ribosomal RNA
SCO: Single-copy ortholog
SINE: Short interspersed nuclear element
SnoRNA: Small nucleolar RNA
snRNA: Small nuclear RNA
SSR: Simple sequence repeat
TE: Transposable element
tRNA: Transfer RNA
TR: Tandem repeat
VL: Visceral leishmaniasis
WDAG: Weighted directed acyclic graph
