# Correction: The genome of *Phlebotomus chinensis*, the primary vector of visceral leishmaniasis in China: insights from chromosome-level assembly and comparative analysis

**DOI:** 10.1186/s40249-026-01430-z

**Published:** 2026-03-26

**Authors:** Haowei Dong, Wenqi Shan, Qiuming Zhou, Hao Yuan, Kang Wang, Yuanyuan Li, Wenbing Zhong, Maimaitijiang Wumaier, Anjie Yang, Huiying Chen, Bing Rui, Yajun Ma, Shizhu Li, Heng Peng

**Affiliations:** 1 Department of Pathogen Biology, College of Basic Medicine, Naval Medical University, Shanghai, 200433 China; 2 Present Address: Key Laboratory of Biosafety Defense, Ministry of Education, Beijing, China; 3 Present Address: Faculty of Naval Medicine, Naval Medical University, Shanghai, 200433 China; 4 Yangquan Center for Disease Control and Prevention, YangquanShanxi, 045000 China; 5 Chinese Center for Disease Control and Prevention, National Institute of Parasitic Diseases, Shanghai, 200025 China; 6 Haikou Center for Disease Control and Prevention, Haikou, 570203 Hainan China; 7 Institute of Parasitic and Brucellosis Prevention and Treatment, Center for Disease Control and Prevention of Xinjiang Uygur Autonomous Region, Urumqi, 830002 China

**Correction: Infectious Diseases of Poverty (2026) 15:20**

 10.1186/s40249-026-01417-w

Following the publication of original article [1], it is reported that Table 1 was accidentally omitted during production. Table 1 has been added back to the article.

The original article [1] has been corrected.
