# Supplementary material for: The genome of Phlebotomus chinensis, the primary vector of visceral leishmaniasis in China: insights from chromosome-level assembly and comparative analysis
Source: Infect Dis Poverty. 2026 Feb 6;15:20. doi: 10.1186/s40249-026-01417-w (PMC12879420; doi:10.1186/s40249-026-01417-w)
Supplement: Supplementary file 2 — Additional file 2. [file 40249_2026_1417_MOESM2_ESM.docx]

**Supplementary Files**

Supplementary Figure S1. Repeat and gene annotation feature of *Ph. chinensis*.

A: Transposon divergence rate of *Ph. chinensis*. Using the total library file as a reference, classification results were obtained via RepeatMasker. The x-axis represents the sequence divergence rate between annotated TE sequences in the genome and their corresponding sequences in the total library file. The y-axis represents the percentage of TE sequences in the genome at each divergence rate. Different TE types are indicated by different colors: DNA (red), LINE (black), LTR (yellow), and SINE (green). B: Venn diagram of protein-coding gene annotations across different databases. Each colored block represents the number of genes annotated by the corresponding database (KOG, SwissProt, NR, KEGG, COG). C: Protein-coding gene features of seven species, including average gene length, CDS length, exon number, exon length, intron number, and intron length. The gene features are largely consistent across the seven species, indicating the accuracy of our annotation.

Supplementary Figure S2. Synteny analysis of protein sequences between *Ph. chinensis* and related sandfly species.

A: Synteny of protein sequence alignment between *Ph. chinensis* and *Lu. longipalpis*. Each block represents a chromosome of the respective species. The overall chromosomal correspondence can be observed, and“the lack of diagonal synteny may be associated with the evolutionary characteristics of the two species. B: Synteny of protein sequence alignment between *Ph. chinensis* and *Ph. papatasi*.
