# Supplementary figures and images for: The genome of Phlebotomus chinensis, the primary vector of visceral leishmaniasis in China: insights from chromosome-level assembly and comparative analysis
Source: Infect Dis Poverty. 2026 Feb 6;15:20. doi: 10.1186/s40249-026-01417-w (PMC12879420; doi:10.1186/s40249-026-01417-w)

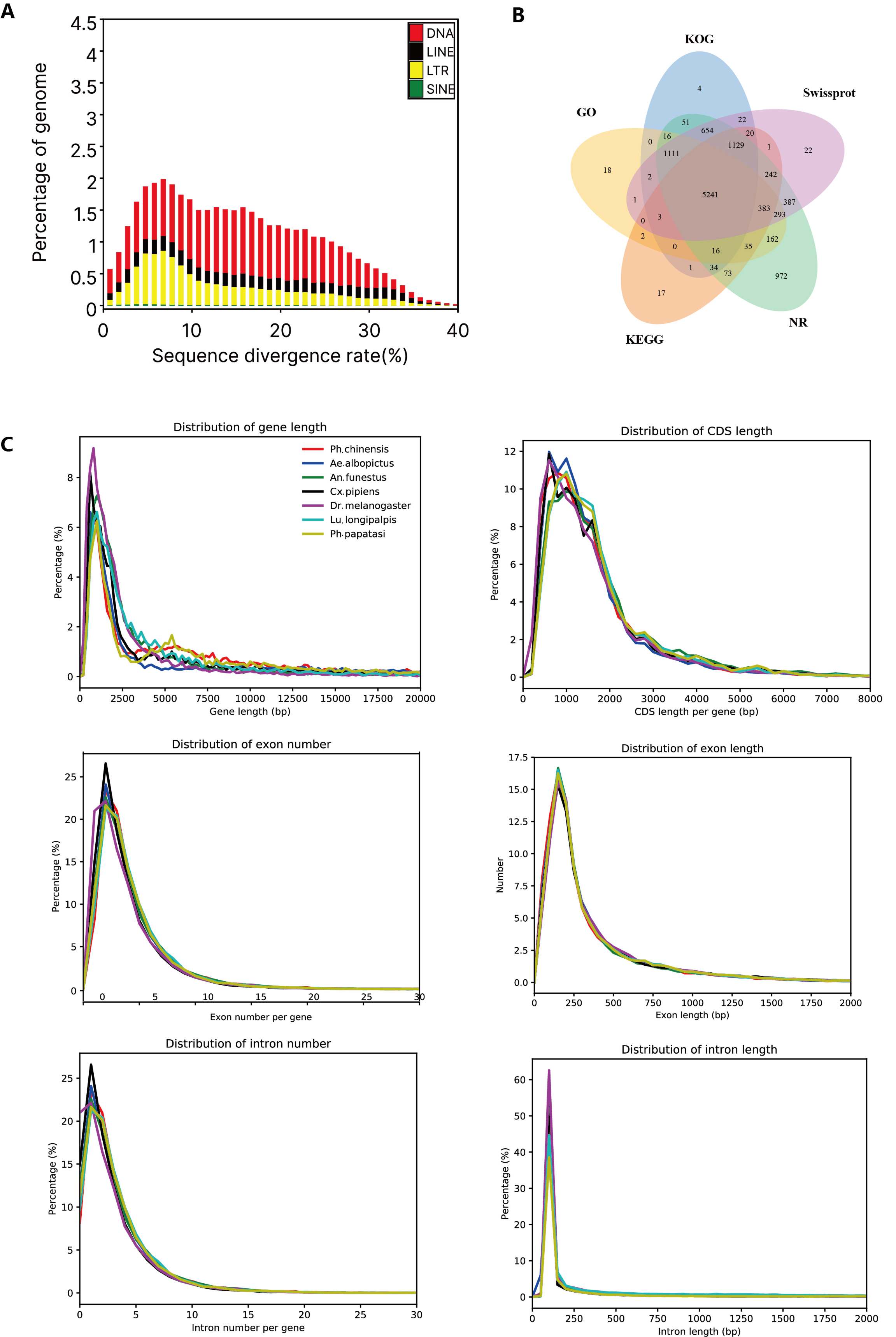

Supplement: Supplementary file 3 — Additional file 3. [file 40249_2026_1417_MOESM3_ESM.jpg]

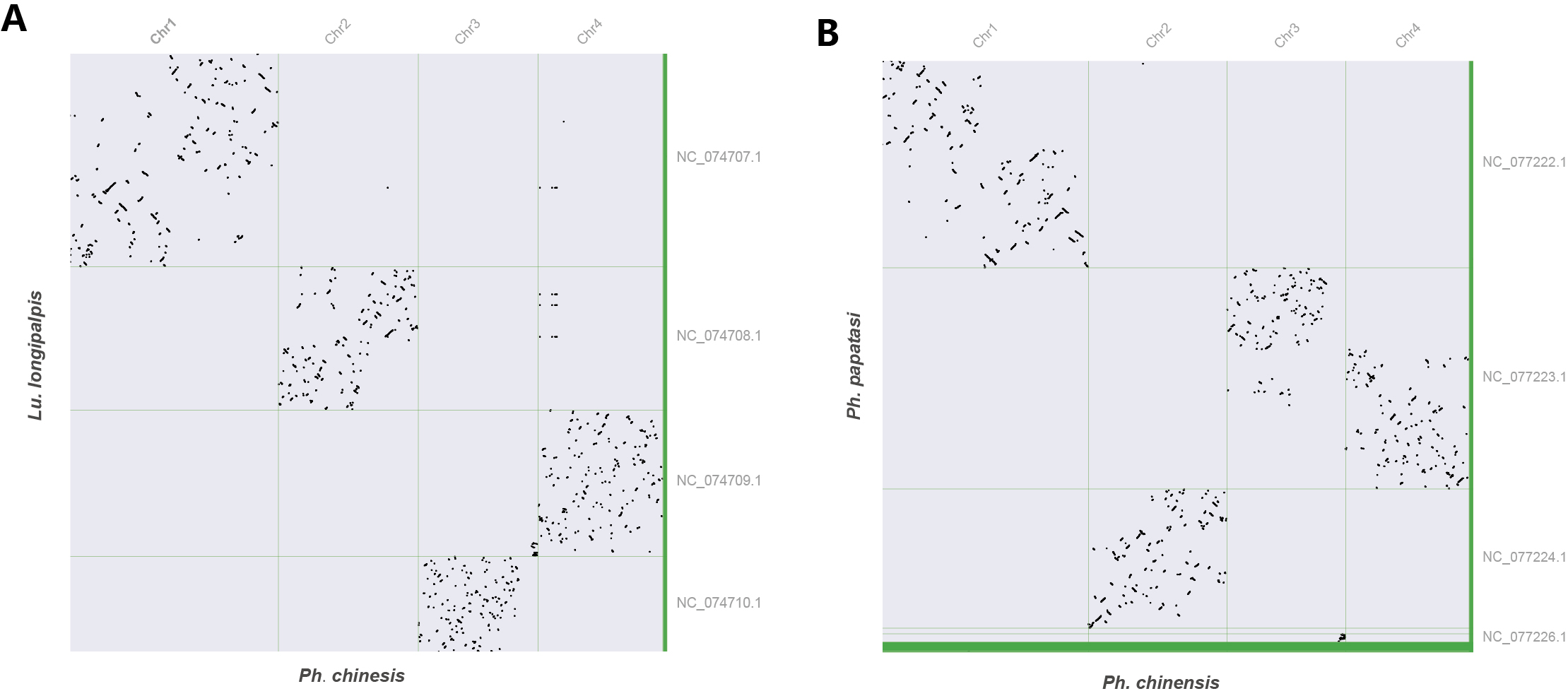

Supplement: Supplementary file 4 — Additional file 4. [file 40249_2026_1417_MOESM4_ESM.jpg]
